# Homoeopathy and the Royal College of Physicians, London

**Published:** 1852-03

**Authors:** 


*Homœopathy and the Royal College of Physicians*, (London —The following letter from the President of the Royal College of Physicians was sent in reply to an application from a homœopathic practitioner for admission to examination for the Degree of Doctor of Medicine:

"Sir:—The foundation of the Royal College of Physicians was for the purpose of guaranteeing to the public skilful and safe practitioners.

"The College of Physicians regard the so-called homœopathists as neither skilful nor safe practitioners.

"Therefore the College cannot, without betraying a sacred trust, give its license to persons whom they regard as wholly unworthy their confidence, and with whom it is not possible they can hold any communion.

"I remain, Sir, your obedient servant,

"JOHN AYRTON PARIS."

